# Risk of recurrence in chronic hepatitis B patients developing hepatocellular carcinoma with antiviral secondary prevention failure

**DOI:** 10.1371/journal.pone.0188552

**Published:** 2017-11-27

**Authors:** I-Cheng Lee, Gar-Yang Chau, Yi-Chen Yeh, Yee Chao, Teh-Ia Huo, Chien-Wei Su, Han-Chieh Lin, Ming-Chih Hou, Yi-Hsiang Huang

**Affiliations:** 1 Division of Gastroenterology and Hepatology, Department of Medicine, Taipei Veterans General Hospital, Taipei, Taiwan; 2 Faculty of Medicine, National Yang-Ming University School of Medicine, Taipei, Taiwan; 3 Institute of Clinical Medicine, National Yang-Ming University, Taipei, Taiwan; 4 Department of Surgery, Taipei Veterans General Hospital, Taipei, Taiwan; 5 Department of Pathology and Laboratory Medicine, Taipei Veterans General Hospital, Taipei, Taiwan; 6 Cancer Center, Taipei Veterans General Hospital, Taipei, Taiwan; 7 Institute of Pharmacology, National Yang-Ming University School of Medicine, Taipei, Taiwan; Icahn School of Medicine at Mount Sinai, UNITED STATES

## Abstract

**Background:**

Nucleos(t)ide analogues (NUCs) treatment can reduce the risk of hepatocellular carcinoma (HCC) development and recurrence in chronic hepatitis B (CHB) patients. However, the risk of recurrence in CHB patients who develop HCC despite NUC treatment remains unclear.

**Methods:**

167 consecutive CHB patients receiving curative resection for HCC with NUC therapy after surgery were retrospectively enrolled. Thirty-eight patients who developed HCC despite NUC therapy for more than 1 year were defined as secondary prevention failure. The other 129 patients started NUC therapy after surgery. Factors associated with recurrence-free survival (RFS) and overall survival (OS) were evaluated.

**Results:**

The 5-year RFS and OS rates were 44.7% and 77.3%, respectively. Sex, BMI, BCLC stage, AFP levels and cirrhosis status were the independent predictors of RFS, while microvascular invasion was the independent predictor of OS. The RFS was comparable between patients with and without NUC secondary prevention. In the subgroup analysis, the RFS was significantly worse in cirrhotic patients with secondary prevention failure (hazard ratio = 2.373, p = 0.009). Secondary prevention failure did not have adverse impact on OS. Among 84 patients with recurrence, 58.3% of the cases remained in BCLC stage A, and 53.6% received a second curative treatment. Long-term NUC therapy may lead to a decline of non-invasive indices of hepatic fibrosis in HCC patients.

**Conclusions:**

In general, the risk of recurrence and survival are comparable between patients with and without secondary prevention failure. However, a higher risk of recurrence was observed in cirrhotic patients with secondary prevention failure.

## Introduction

Despite the improvement in controlling risk factors and surveillance, hepatocellular carcinoma (HCC) remains the third leading cause of cancer-related deaths in the world [1]. Chronic hepatitis B virus (HBV) infection is the major cause of HCC worldwide, accounting for 50%-80% of global HCC cases [2]. Universal immunization against HBV has successfully reduced the incidence of HBV in younger generations, and is the primary preventive strategy for HBV-related HCC (primary prevention) [3]. However, there are still about 250 million HBV carriers worldwide, and these patients have a significantly increased risk of HCC, especially in patients with cirrhosis [4].

Nucleos(t)ide analogues (NUCs), which may suppress viral replication, attenuate the progression of liver disease, and reverse liver fibrosis and cirrhosis, are the mainstay of the treatment for chronic hepatitis B (CHB) [5,6]. In addition, NUC treatment has been reported to attenuate 52%-78% of the risk of HCC development in patients with CHB, and thus may be adopted as a secondary preventive strategy for HBV-related HCC (secondary prevention) [7,8]. Nevertheless, despite long-term NUC therapy, HCC risk could not be completely eliminated [9,10]. The annual HCC incidences rate after NUC treatment ranges from 0.01% to 1.4% in non-cirrhotic patients, and from 0.9% to 5.4% in those with cirrhosis [11,12,13]. Older age, male gender, advanced liver disease, diabetes mellitus (DM) and not achieving virological response (VR) have also been shown to be predictors of HCC development in CHB patients under NUC therapy [12,14,15].

In patients who developed HCC at an early stage, hepatic resection, liver transplantation, and radiofrequency ablation are considered potentially curative treatment [16]. However, the long-term outcome of HCC is still unsatisfactory even after curative treatment, and approximately 70% of patients will develop tumor recurrence within 5 years after curative resection [16,17,18]. In HBV-related HCC, HBV viral loads and viral mutations are important risk factors for tumor recurrence after a hepatic resection [19,20], and recent studies suggest that NUCs treatment after a curative resection for HBV-related HCC is associated with a reduced risk of recurrence and may prolong survival [21,22,23,24,25,26]. Therefore, NUCs therapy could be a tertiary preventive strategy (tertiary prevention), and is now widely prescribed for patients with HBV-related HCC after curative resection.

CHB patients could develop HCC even under NUC therapy (secondary prevention failure). It is unclear whether the outcome of these patients was different from those without NUC secondary prevention. The aim of this study was to evaluate factors associated with the recurrence and survival of patients with HBV-related HCC receiving NUC tertiary prevention after curative resection, and to compare the outcomes in patients with and without NUC secondary prevention.

## Material and methods

### Patients

From October 1, 2007 to May 31, 2014, 516 consecutive patients receiving a surgical resection for HBV-related HCC in Taipei Veterans General Hospital were retrospectively screened for the status of NUC therapy after surgery (S1 Fig). Exclusion criteria included hepatitis C virus co-infection, other malignancy, presence of extrahepatic metastasis, surgical mortality, non-curative resection, NUC starting more than 1 year after surgery or with a duration of less than 90 days, uncertain NUC usage or duration, poor virological response to NUC therapy at the time of surgery (HBV DNA >2000 IU/mL), or lost to follow-up after surgery. All participants received standard of care treatment. Patients undergoing NUC therapy for more than 1 year before HCC development and who continued NUC therapy after their curative resection were defined as the secondary prevention failure group (n = 38), whereas patients without NUC therapy before the development of HCC and who started NUC within 1 year after their curative resection were defined as the tertiary prevention group (n = 129). Patients who started NUC therapy before HCC development fulfilled the treatment criteria for CHB according to the APASL treatment guidelines [27]. The selection of NUC depended on the preference of the caring physicians. This study was approved by the Institutional Review Board, Taipei Veterans General Hospital, which complied with standards of the Declaration of Helsinki and current ethical guidelines. Due to the retrospective nature of the study, the Institutional Review Board waived the need for written informed consent. The identifying information of the enrolled subjects has been delinked and therefore authors could not access to these information.

The diagnosis of HCC and resectability were assessed before surgery by contrast-enhanced computed tomography (CT) or magnetic resonance imaging (MRI), which fulfilled the diagnostic criteria of the American Association for the Study of Liver Diseases (AASLD) treatment guidelines for HCC [28], and were confirmed pathologically after surgery. Curative surgical resection was confirmed by contrast-enhanced CT or MRI after surgery. Patients were followed every 2–3 months with measurement of serum AFP, ultrasonography, CT or MRI, with the mean interval of follow-up 84.8 days after the surgery. Tumor recurrence was suspected in the presence of elevation of serum alpha-fetoprotein (AFP) levels and ultrasonography detection of a new hepatic lesion, and was confirmed by contrast-enhanced CT or MRI.

### Endpoint

The primary endpoint was recurrence-free survival (RFS), defined as the time from surgical resection to tumor recurrence confirmed by contrast-enhanced CT or MRI. The secondary endpoint was overall survival (OS), defined as the time from surgical resection to death, and liver fibrosis regression, as determined by paired liver pathology and non-invasive indices of hepatic fibrosis.

### Biochemistry, virological tests, and histological features

The following clinical features and biochemistry were collected for analysis: age, sex, diabetes mellitus (DM) status, Barcelona Clinic Liver Cancer (BCLC) stage, Child-Pugh score, serum AFP, alanine aminotransferase (ALT), aspartate aminotransferase (AST), creatinine, albumin, total bilirubin levels, platelet count, and prothrombin time (measured by international normalized ratio [INR]). Serum HBeAg and serum AFP were measured by radio-immunoassay kits (Abbott Laboratories, North Chicago, IL and Serono Diagnostic SA, Coinsin/VD, Switzerland, respectively). Serum biochemistry tests were measured by systemic multi-autoanalyzer (Technicon SMAC, Technicon Instruments Corp., Tarrytown, NY). HBV DNA was determined by Roche Cobas Tagman HBV DNA assay (detection limit of 12 IU/mL, Roche Diagnostics, Switzerland). HBsAg levels were quantified using the Elecsys HBsAg II assay (detection limit of 0.05 IU/mL, Roche Diagnostics GmbH, Mannheim, Germany).

An Albumin-Bilirubin (ALBI) grade was calculated using the formula: linear predictor = (log_10_ bilirubin x 0.66) + (albumin x 0.085), where bilirubin is in umol/L and albumin in g/L; and the cut points of the ALBI grade were as follows: xb ≤-2.60 (ALBI grade 1), more than -2.60 to ≤-1.39 (ALBI grade 2), and xb more than -1.39 (ALBI grade 3) [29]. Two non-invasive indices of hepatic fibrosis, the fibrosis-4 index (FIB-4) and AST to platelet ratio index (APRI), were selected to compare the status of hepatic fibrosis after NUC treatment. FIB-4 was calculated using the formula: Age (years) x AST [U/L]/platelet count [10^9^/L] x (ALT [U/L])^1/2^, while APRI was calculated using the formula: ([AST/ULN]/platelet count [10^9^/L]) x 100 [30,31]. FIB-4 and APRI were calculated at the baseline, year 2 and year 5 for all patients with available data.

Histological features of tumors and non-tumor liver tissue, including tumor size, tumor number, microvascular invasion, tumor capsule integrity, surgical safe margin, status of steatosis, and cirrhosis (defined as Ishak fibrosis score 5–6 [32]) were recorded.

### Statistical analysis

Values were expressed as median (ranges) or as mean ± standard deviation when appropriate. The Mann-Whitney *U* test was used to compare continuous variables. Pearson chi-square analysis or the Fisher exact test were used to compare categorical variables. The Wilcoxon signed ranks test was used to compare serial changes in the FIB-4 score. The Kaplan-Meier method was used to estimate survival rates. The log-rank test was used to compare survival curves between patient groups. Analysis of prognostic factors for survival was performed using the Cox proportional hazards model. Variables that achieved statistical significance (*p*<0.05) or those close to significance (*p*<0.1) by univariate analysis were subsequently included in the multivariate analysis. A two-tailed *p*<0.05 was considered statistically significant. All statistical analyses were performed using the Statistical Package for Social Sciences (SPSS 17.0 for Windows, SPSS Inc, Chicago, IL).

## Results

The baseline characteristics of 167 HCC patients are shown in Table 1. The mean age was 58.7 years, 91.9% were males, 20.4% were HBeAg-positive, and 51.7% had histological cirrhosis. The majority of patients (89%) were Child-Pugh score 5, and 64.1% were BCLC stage A. Compared with the tertiary prevention group, patients in the secondary prevention failure group had significantly lower HBV viral loads, ALT, AST levels, smaller tumor size, and earlier BCLC stage. Before surgery, 86.8% of patients who received NUC secondary prevention had undetectable HBV DNA, whereas in the tertiary prevention group, only 2 (1.7%) patients had undetectable HBV DNA and 8 (6.7%) patients HBV DNA <2000 IU/mL.

**Table 1:** Baseline characteristics of the 167 HCC patients receiving NUC therapy after curative resection.

	Overall (n = 167)	Secondary prevention failure group (n = 38, 22.8%)	Tertiary prevention group (n = 129, 77.2%)	*p*
Age (years)	58.6 ± 11.4	58.2 ± 11.6	58.7 ± 11.4	0.668
Sex (male), n (%)	154 (92.2)	36 (94.7)	118 (91.5)	0.735
BMI (kg/m^2^)	24.7 ± 3.3	24.3 ± 3.3	24.8 ± 3.4	0.347
BMI >27.5 kg/m^2^	34 (20.4)	6 (17.6)	28 (21.7)	0.557
BMI >30 kg/m^2^	10 (6)	0 (0)	10 (7.8)	0.119
Diabetes, n (%)	37 (22.2)	9 (23.7)	28 (21.7)	0.971
Child-Pugh score 5/6/7, n (%)	149/17/1 (89.2/10.2/0.6)	36/2/0 (94.7/5.3/0)	113/15/1 (87.6/11.6/0.8)	0.443
HBV DNA (Log IU/mL)*	4.56 ± 2.11	1.89 ± 1.64	5.37 ± 1.47	<0.001
Undetectable HBV DNA, n (%)	35 (22.4)	33 (94.3)	2 (5.7)	<0.001
HBV DNA <2000 IU/mL	46 (29.3)	38 (100)	8 (6.7)	<0.001
HBsAg <200 IU/mL, n (%)*	26 (19)	8 (29.6)	18 (16.4)	0.193
HBeAg-positive, n (%)	31 (19.1)	6 (15.8)	25 (20.2)	0.716
BCLC stage A/B/C, n (%)	107/53/7 (64.1/31.7/4.2)	31/4/3 (81.6/10.5/7.9)	76/49/4 (58.9/38/3.1)	0.002
Tumor size (cm)	4.36 ± 3.05	3.20 ± 2.31	4.7 ± 3.2	0.001
Multiple tumors, n (%)	32 (19.2)	5 (15.6)	27 (20.9)	0.403
AFP (ng/mL)	23.2 (1.2–67467)	13.5 (1.2–18873)	26.5 (1.5–67467)	0.491
Albumin (g/dL)	4.10 ± 0.43	4.17 ± 0.39	4.08 ± 0.45	0.463
Total bilirubin (mg/dL)	0.87 ± 0.42	0.84 ± 0.44	0.86 ± 0.39	0.564
Albumin-Bilirubin (ALBI) score	-2.74 ± 0.38	-2.80 ± 0.36	-2.72 ± 0.39	0.478
ALBI grade I/II, n (%)	112/55 (67.1/32.9)	29/9 (76.3/23.7)	83/46 (64.3/35.7)	0.236
Platelet count (10^9^/L)	161 ± 64	163 ± 64	161 ± 64	0.271
Prothrombin time (INR)	1.08 ± 0.07	1.08 ± 0.07	1.08 ± 0.07	0.968
Creatinine (mg/dL)	0.93 ± 0.22	0.90 ± 0.18	0.93 ± 0.24	0.596
ALT (U/L)	60 ± 57	32 ± 10	68 ± 63	<0.001
AST (U/L)	53 ± 41	33 ± 14	59 ± 45	0.003
**Histological features, n (%)**				
Microvascular invasion	120 (71.9)	24 (63.2)	96 (74.4)	0.250
Intact tumor capsule	45 (26.9)	11 (24.4)	34 (26.4)	0.914
Presence of steatosis	67 (48.2)	15 (44.1)	52 (49.5)	0.726
Surgical safe margin >1 cm, n (%)*	51 (34.7)	13 (35.1)	38 (34.5)	1.000
Histological cirrhosis, n (%)	84 (50.3)	16 (42.1)	68 (52.7)	0.335
NUC type, n (%): Low / high genetic barrier**				
Before surgery (Secondary prevention)	-	7/31 (18.4/81.6)	-	-
After surgery (Tertiary prevention)	15/152 (9.0/91.0)	5/33 (13.2/86.8)	10/119 (7.8/92.2)	0.336
Undetectable HBV DNA within 1 year after surgery, n (%)*	140 (87.5)	35 (94.6)	105 (85.4)	0.166

*Available baseline HBV DNA data: n = 156; available HBsAg data: n = 137; available safe margin distance: n = 147; available virological response status: n = 160.

** Low genetic barrier: lamivudine, adefovir, telbivudine; high genetic barrier: entecavir, tenofovir.

The median duration of NUC therapy in the secondary prevention failure group was 20.8 months before the development of HCC. The majority of patients received either entecavir or tenofovir. All but 3 cases in the secondary prevention failure group and 85.4% of patients in tertiary prevention group achieved undetectable HBV DNA within 1 year after the surgery.

### Factors associated with recurrence-free survival (RFS) and early recurrence

**Fig 1:**
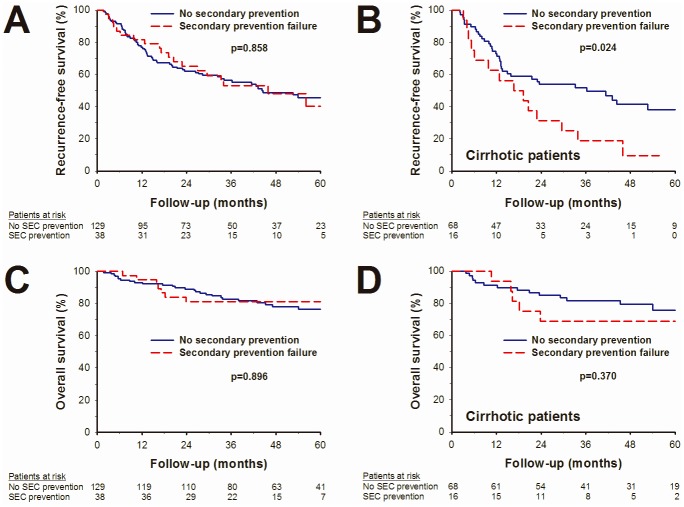
Kaplan—Meier curves of recurrence-free survival (RFS) and overall survival (OS). (A) RFS in patients with and without NUC secondary prevention. (B) RFS in cirrhotic patients with and without NUC secondary prevention. (C) OS in patients with and without NUC secondary prevention. (D) OS in cirrhotic patients with and without NUC secondary prevention.

After a median follow-up of 45.3 months, 84 patients developed HCC recurrence. The estimated 1-, 3- and 5-year RFS rates were 78%, 55.4% and 44.7%, respectively. The median RFS was 45.9 months in the secondary prevention failure group, and 44.3 months in the tertiary prevention group (p = 0.858, Fig 1A). In univariate analysis, the factors associated with RFS included sex, BMI, BCLC stage, tumor number, serum AFP and AST levels, surgical safe margin and histological cirrhosis (Table 2). By multivariate analyses, independent predictors of RFS were female sex (HR = 0.197, p = 0.024), BMI >27.5 kg/m^2^ (hazard ratio (HR) = 1.882, p = 0.012), BCLC stage B-C (HR = 1.573, p = 0.046), serum AFP level >20 ng/mL (HR = 2.082, p = 0.001) and histological cirrhosis (HR = 2.257, p<0.001). Baseline viral loads, HBsAg levels, HBeAg status, NUC type, and one-year virological response were not associated with RFS.

**Table 2:** Univariate and multivariate analyses of factors associated with recurrence-free survival.

		Univariate			Multivariate		
		HR	95% CI	*P*	HR	95% CI	*P*
Age (years)	>60 vs ≤60	1.170	0.762–1.787	0.472			NA
Sex	Female vs male	0.215	0.053–0.876	0.032	0.197	0.048–0.808	0.024
BMI (kg/m^2^)	>27.5 vs ≤27.5	1.739	1.076–2.813	0.024	1.882	1.152–3.074	0.012
Diabetes	Yes vs no	1.030	0.611–1.735	0.913			NA
Child-Pugh score	6–7 vs 5	1.116	0.576–2.162	0.745			NA
BCLC stage	B-C vs A	1.661	1.076–2.565	0.022	1.573	1.007–2.455	0.046
HBV DNA (IU/mL)	>20 vs ≤20	1.108	0.648–1.894	0.709			NA
	>200 vs ≤200	0.951	0.577–1.567	0.845			NA
HBsAg (IU/mL)	>200 vs ≤200	1.052	0.574–1.928	0.870			NA
HBeAg	Positive vs negative	0.976	0.565–1.685	0.931			NA
NUC secondary prevention failure	Yes vs no	1.047	0.633–1.731	0.859			NA
NUC type	High genetic barrier vs low genetic barrier	1.785	0.771–4.132	0.176			NA
Undetectable HBV DNA within 1 year after surgery	Yes vs no	1.320	0.633–2.752	0.459			NA
Tumor size (cm)	>5 vs ≤5	1.446	0.914–2.288	0.115			NA
Tumor number	Multiple vs single	2.158	1.319–3.533	0.002			NS
AFP (ng/mL)	>20 vs ≤20	1.750	1.131–2.708	0.012	2.082	1.333–3.253	0.001
Bilirubin (mg/dL)	>1.2 vs ≤1.2	1.388	0.779–2.474	0.266			NA
Albumin (g/dL)	>3.5 vs ≤3.5	0.770	0.385–1.539	0.460			NA
ALBI grade	Every 1 grade	1.514	0.973–2.354	0.066			NS
Creatinine (mg/dL)	>1.2 vs ≤1.2	1.098	0.529–2.280	0.801			NA
Prothrombin time (INR)	>1.1 vs ≤1.1	1.545	0.975–2.448	0.064			NS
Platelet count (10^9^/L)	>100 vs ≤100	0.600	0.351–1.026	0.062			NS
ALT (U/L)	>80 vs ≤80	0.697	0.377–1.289	0.250			NA
AST (U/L)	>80 vs ≤80	1.572	0.911–2.714	0.104			NA
Microvascular invasion	Yes vs no	1.330	0.817–2.166	0.251			NA
Incomplete tumor capsule	Yes vs no	1.180	0.725–1.919	0.506			NA
Safe margin >1 cm	Yes vs no	0.523	0.307–0.891	0.017			NS
Presence of steatosis	Yes vs no	0.931	0.572–1.516	0.774			NA
Histological cirrhosis	Presence vs absence	2.147	1.378–3.344	0.001	2.257	1.441–3.534	<0.001

HR, hazard ratio; CI, confidence interval; NA, not adopted; NS, not significant; ALBI, Albumin-Bilirubin.

Among the 84 patients with HCC recurrence, 60 (71.4%) developed early recurrence within 2 years, while 24 (28.6%) had late recurrence after 2 years of surgery. By multivariate analyses, independent predictors of early recurrence in the overall cohort were BMI >27.5 kg/m^2^ (HR = 2.185, p = 0.010), BCLC stage B-C (HR = 2.526, p = 0.001), serum AFP level >20 ng/mL (HR = 2.212, p = 0.006) and histological cirrhosis (HR = 2.989, p<0.001) (S1 Table). Independent predictors of late recurrence after 2 years of surgery in patients without early recurrence were multiple tumors (HR = 3.028, p = 0.030), and INR >1.1 (HR = 3.359, p = 0.004) (S2 Table).

### Subgroup analysis for the association of NUC secondary prevention failure and HCC recurrence

**Fig 2:**
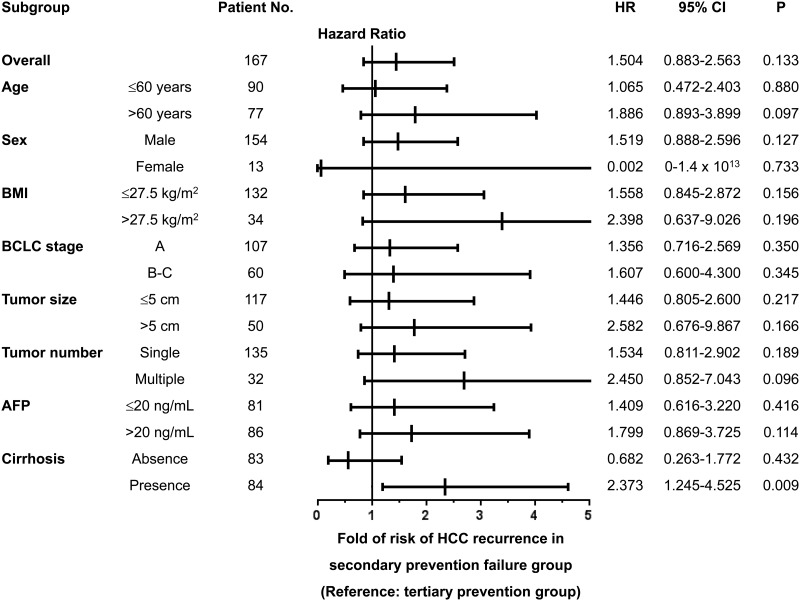
Multivariable stratified analyses for the association of NUC secondary prevention failure and HCC recurrence. The relative risk of recurrence in secondary prevention group was compared to the tertiary prevention group. Adjusted factors include age, sex, BMI, BCLC stage, AFP and cirrhosis. HR, hazard ratio; CI, confidence interval.

Due to the different baseline characteristics in tumor stage, size, liver function and HBV viral load between NUC secondary prevention failure and tertiary prevention groups, a multi-variable stratified subgroup analysis for the association of secondary prevention failure with the risk of tumor recurrence according to baseline prognostic factors was performed. As shown in Fig 2, the risk of HCC recurrence was comparable between the NUC secondary prevention failure and tertiary prevention groups in most of the sub-analysis. Note worthily, in subgroup patients with histological cirrhosis, there was a significantly higher risk of HCC recurrence in patients with NUC secondary prevention failure as compared with the tertiary prevention group (HR = 2.373, p = 0.009, Figs 1B and 2). By multivariate analysis, the NUC secondary prevention failure remains an independent predictor of poor RFS in cirrhotic patients (HR = 2.346, p = 0.010, Table 3).

**Table 3:** Univariate and multivariate analyses of factors associated with recurrence-free survival in cirrhotic patients.

		Univariate			Multivariate		
		HR	95% CI	*P*	HR	95% CI	*P*
Age (years)	>60 vs ≤60	0.984	0.570–1.697	0.953			NA
Sex	Female vs male	0.343	0.047–2.491	0.290			NA
BMI (kg/m^2^)	>27.5 vs ≤27.5	1.821	0.997–3.325	0.051			NS
Diabetes	Yes vs no	1.622	0.846–3.113	0.146			NA
Child-Pugh score	6–7 vs 5	0.861	0.366–2.029	0.733			NA
BCLC stage	B-C vs A	1.609	0.913–2.834	0.100			NA
HBV DNA (IU/mL)	>200 vs ≤200	0.556	0.300–1.028	0.061			NS
HBsAg (IU/mL)	>200 vs ≤200	1.238	0.572–2.679	0.588			NA
HBeAg	Positive vs negative	0.716	0.359–1.431	0.345			NA
NUC secondary prevention failure	Yes vs no	2.018	1.085–3.753	0.027	2.346	1.223–4.501	0.010
NUC type	High genetic barrier vs low genetic barrier	0.968	0.300–3.119	0.957			NA
Undetectable HBV DNA within 1 year after surgery	Yes vs no	0.882	0.373–2.088	0.776			NA
Tumor size (cm)	>5 vs ≤5	1.274	0.679–2.391	0.450			NA
Tumor number	Multiple vs single	1.459	0.808–2.636	0.210			NA
AFP (ng/mL)	>20 vs ≤20	2.106	1.211–3.663	0.008	2.309	1.281–4.162	0.005
Bilirubin (mg/dL)	>1.2 vs ≤1.2	1.046	0.508–2.154	0.902			NA
Albumin (g/dL)	>3.5 vs ≤3.5	0.806	0.319–2.034	0.647			NA
ALBI grade	Every 1 grade	1.097	0.629–1.915	0.744			NA
Creatinine (mg/dL)	>1.2 vs ≤1.2	0.386	0.120–1.240	0.110			NA
Prothrombin time (INR)	>1.1 vs ≤1.1	1.388	0.791–2.435	0.253			NA
Platelet count (10^9^/L)	>100 vs ≤100	0.888	0.496–1.591	0.690			NA
ALT (U/L)	>80 vs ≤80	0.464	0.197–1.094	0.079			NS
AST (U/L)	>80 vs ≤80	0.922	0.532–1.599	0.772			NA
Microscopic vascular invasion	Yes vs no	1.348	0.729–2.494	0.341			NA
Incomplete tumor capsule	Yes vs no	1.008	0.537–1.890	0.981			NA
Presence of steatosis	Yes vs no	0.946	0.517–1.728	0.856			NA
Safe margin >1 cm	Yes vs no	0.468	0.218–1.006	0.052	0.407	0.186–0.889	0.024

HR, hazard ratio; CI, confidence interval; NA, not adopted; NS, not significant; ALBI, Albumin-Bilirubin.

### Tumor stage and rescue therapy at the time of first recurrence

**Fig 3:**
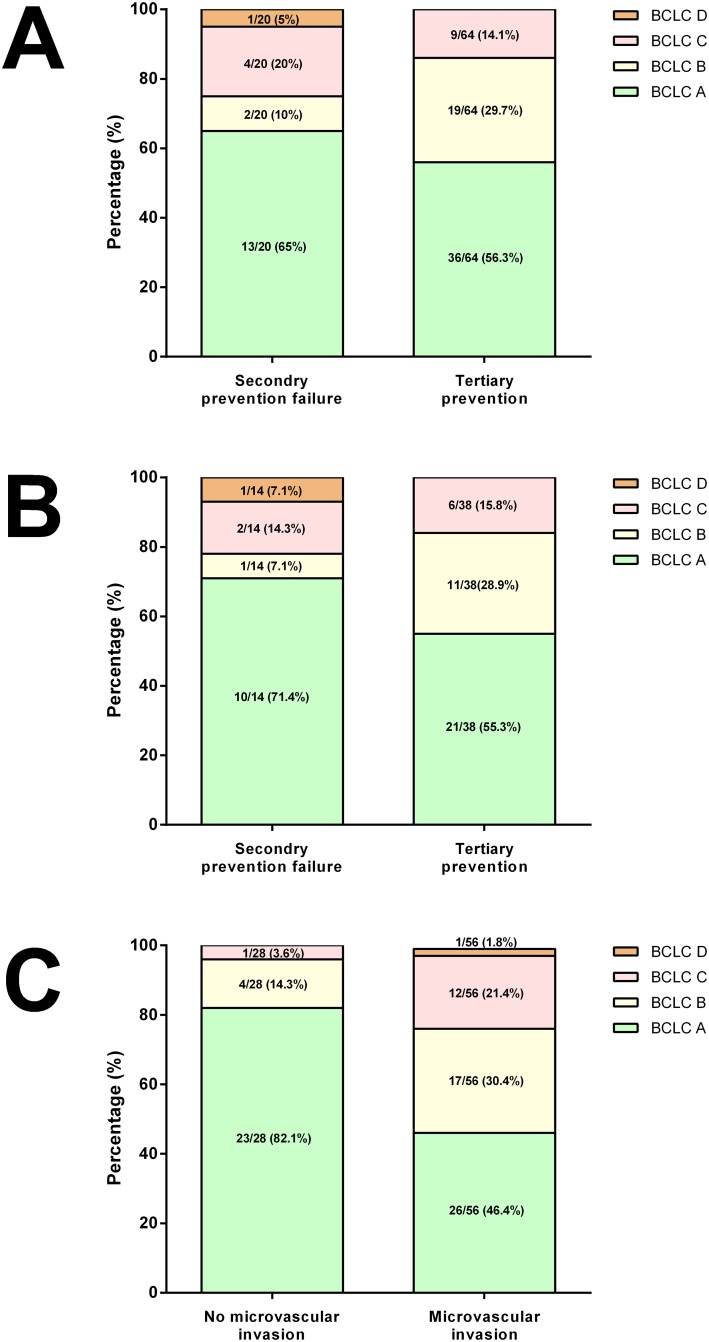
Tumor stage at the time of first HCC recurrence. (A) Recurrence stage in patients with and without NUC secondary prevention (p = 0.724). (B) Recurrence stage in cirrhotic patients with and without NUC secondary prevention (p = 0.895). (C) Recurrence stage in patients with and without microvascular invasion (p = 0.002).

Among the 84 patients with HCC recurrence, 49 patients (58.3%) remained in BCLC stage A, while 21 (25%), 13 (15.5%) and 1 (1.2%) patients progressed to BCLC stage B, C and D, respectively. The tumor recurrence stage was comparable between patients with and without NUC secondary prevention (p = 0.724, Fig 3A). A similar HCC recurrence stage migration was observed in 52 patients with cirrhosis, and the recurrence stage was also comparable between patients with and without NUC secondary prevention (p = 0.895, Fig 3B).

Forty-five patients (53.6%) received a second curative treatment after the first HCC recurrence, including 10 patients (11.9%) receiving a second curative resection, and 35 patients (41.7%) receiving local ablation therapy. Only four patients (4.8%) did not receive HCC treatment after recurrence, including three patients who refused further treatment and one BCLC-D patient with hepatic decompensation due to post-operative bile leakage.

### Factors associated with overall survival (OS)

Thirty five cases died during follow-up, including 25 (71.4%) due to tumor progression, 1 (2.9%) due to hepatic compensation without apparent tumor recurrence, 4 (11.4%) due to treatment-related complications, 3 (8.6%) due to infection and 2 (5.7%) with unknown cause. The estimated 1-, 3- and 5-year OS rates were 93.4%, 82.4% and 77.3%, respectively. In univariate analysis, BCLC stage, tumor size, serum AST levels, microvascular invasion and surgical safe margin were factors associated with OS (Table 4). NUC secondary prevention failure was not associated with OS in the overall patients (p = 0.896, Fig 1C), as well as in subgroup patients with cirrhosis (p = 0.370, Fig 1D). By multivariate analyses, the only independent predictor of OS was microvascular invasion (HR = 8.307, p = 0.041). Patients without microvascular invasion had a significantly earlier recurrent tumor stage as compared to those with microvascular invasion (82.1% vs 46.4%, p = 0.002, Fig 3C).

**Table 4:** Univariate and multivariate analyses of factors associated with overall survival.

		Univariate			Multivariate		
		HR	95% CI	*P*	HR	95% CI	*P*
Age (years)	>60 vs ≤60	1.360	0.693–2.667	0.371			NA
Sex	Female vs male	0.686	0.164–2.864	0.605			NA
BMI (kg/m^2^)	>27.5 vs ≤27.5	1.160	0.525–2.564	0.713			NA
Diabetes	Yes vs no	1.212	0.548–2.677	0.635			NA
Child-Pugh score	6–7 vs 5	1.069	0.376–3.036	0.901			NA
BCLC stage	B-C vs A	2.556	1.298–5.034	0.007			NS
HBV DNA (IU/mL)	>20 vs ≤20	1.043	0.452–2.405	0.922			NA
	>200 vs ≤200	0.997	0.449–2.2158	0.995			NA
HBsAg (IU/mL)	>200 vs ≤200	0.738	0.295–1.850	0.517			NA
HBeAg	Positive vs negative	0.379	0.116–1.240	0.109			NA
NUC secondary prevention failure	Yes vs no	0.946	0.411–2.176	0.896			NA
NUC type	High genetic barrier vs low genetic barrier	1.130	0.343–3.723	0.841			NA
Undetectable HBV DNA within 1 year after surgery	Yes vs no	0.883	0.304–2.564	0.819			NA
Tumor size (cm)	>5 vs ≤5	2.743	1.399–5.378	0.003	2.004	0.955–4.204	0.066
Tumor number	Multiple vs single	1.789	0.833–3.843	0.136			NA
AFP (ng/mL)	>20 vs ≤20	1.865	0.923–3.769	0.083			NS
Bilirubin (mg/dL)	>1.2 vs ≤1.2	1.320	0.546–3.193	0.538			NA
Albumin (g/dL)	>3.5 vs ≤3.5	0.715	0.252–2.031	0.529			NA
ALBI grade	Every 1 grade	1.237	0.612–2.501	0.554			NA
Creatinine (mg/dL)	>1.2 vs ≤1.2	1.454	0.511–4.135	0.483			NA
Prothrombin time (INR)	>1.1 vs ≤1.1	1.164	0.556–2.437	0.687			NA
Platelet count (10^9^/L)	>100 vs ≤100	1.020	0.394–2.643	0.967			NA
ALT (U/L)	>80 vs ≤80	1.344	0.606–2.981	0.467			NA
AST (U/L)	>80 vs ≤80	2.368	1.131–4.958	0.022			NS
Microvascular invasion	Yes vs no	4.520	1.381–14.795	0.013	8.307	1.093–63.118	0.041
Incomplete tumor capsule	Yes vs no	2.365	0.915–6.113	0.076			NS
Safe margin >1 cm	Yes vs no	0.293	0.102–0.843	0.023			NS
Presence of steatosis	Yes vs no	0.662	0.314–1.396	0.279			NA
Histological cirrhosis	Presence vs absence	1.399	0.710–2.759	0.332			NA

HR, hazard ratio; CI, confidence interval; NA, not adopted; NS, not significant; ALBI, Albumin-Bilirubin.

### Liver fibrosis regression in HCC patients receiving NUC therapy

**Fig 4:**
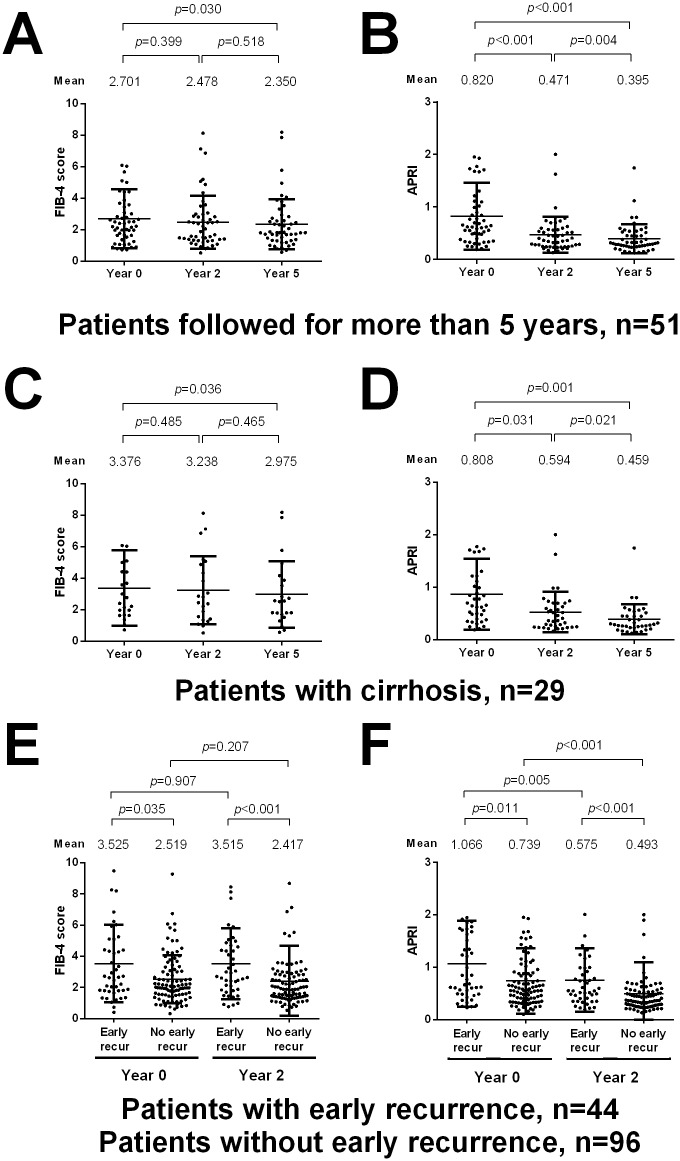
Change of FIB-4 scores and APRI in HCC patients receiving NUCs after curative resection. (A) FIB-4 scores in patients who were followed for more than 5 years. (B) APRI in patients who were followed for more than 5 years. (C) FIB-4 scores in cirrhotic patients who were followed for more than 5 years. (D) APRI in cirrhotic patients who were followed for more than 5 years. (E) FIB-4 scores in patients with and without early recurrence within 2 years. (F) APRI in patients with and without early recurrence within 2 years.

Ten patients received a second curative resection after HCC recurrence, allowing paired comparison of liver histology of the non-tumor part. Among the 10 patients, 5 had fibrosis regression, 3 had a stable Ishak fibrosis stage, and 2 had fibrosis progression (Table 5). In 140 patients who were followed for more than 2 years, the FIB-4 scores were not significantly different between year 0 and year 2 (p = 0.313, S2A Fig), whereas a significant decline of APRI was observed at year 2 (p<0.001, S2B Fig). In 51 patients who were followed for more than 5 years, there were significant declines in both FIB-4 score (p = 0.030, Fig 4A) and APRI (p<0.001, Fig 4B) from the baseline to year 5. Similar declining patterns of FIB-4 scores and APRI were also observed in subgroup patients with cirrhosis (Fig 4C and 4D), as well as in patients with and without NUC secondary prevention (S3 Fig).

**Table 5:** Change of Ishak inflammatory grade and fibrosis score in 10 patients receiving a second hepatic resection for recurrent HCC.

		Ishak inflammatory grade		Ishak fibrosis stage	
Case	Operation interval (mo)	First operation	Second operation	First operation	Second operation
1	34.5	2	3	1	2
2	16.0	3	3	5	5
3	35.7	3	3	6	5
4	28.7	5	4	3	5
5	4.6	6	4	5	4
6	33.6	3	3	3	1
7	24.7	3	3	6	5
8	78.4	3	4	3	2
9	46.7	3	3	4	4
10	21.0	3	3	4	4

As compared to patients without early recurrence within 2 years, patients with early recurrence had significantly higher baseline FIB-4 scores and APRI (Fig 4E and 4F), although there was no significant decline of FIB-4 scores at year 2 in either patients with and without early recurrence (Fig 4E). Interestingly, a significant decrease in APRI at year 2 in both patients with and without early recurrence was observed, and APRI remained significantly higher at year 2 in patients with early recurrence (Fig 4F).

We also examined the correlation between the cirrhosis status and FIB-4 scores and APRI at the baseline. The area under the receiver operating characteristic (AUROC) of FIB-4 and APRI for diagnosing cirrhosis was 0.692 and 0.644 among the overall patients, 0.761 and 0.739 in secondary prevention failure group, 0.664 and 0.603 in the tertiary prevention group, respectively.

## Discussion

Recent studies have shown that antiviral therapy in patients with HBV-related HCC after curative treatment may decrease the risk of HCC recurrence and improve overall survival [22,23,24]. However, previous studies regarding NUC tertiary prevention excluded patients undergoing NUC therapy before curative treatment. Therefore, the clinical significance of maintaining NUC treatment in NUC secondary prevention failure on HCC outcomes remains unclear. In addition, the underlying mechanism of HCC development despite NUC secondary prevention is poorly understood, and whether these patients had different tumor aggressiveness and outcomes remains uncertain. In this study, we found that in HCC patients undergoing NUC treatment after the surgery, the clinical outcomes were comparable between those with and without prior NUC secondary prevention. However, cirrhosis patients with NUC secondary prevention failure had a higher risk of recurrence.

As compared to HCC patients without prior antiviral therapy, patients who developed HCC despite NUC secondary prevention had significantly lower HBV viral loads, lower hepatitis activity, smaller tumor size, and earlier tumor stages at the time of surgical resection. Since patients under NUC therapy may receive more stringent monitoring and HCC surveillance, HCC could be detected at an earlier stage. The 5-year RFS rate of 44.7%% in this study was consistent with previous reports of HCC patients receiving NUC therapy after surgery [22,23]. Sex, BMI, tumor stage, tumor number, serum AFP levels, surgical safe margin and cirrhosis status were predictors of RFS in univariate analysis. The male gender has long been known to enhance the risk for HBV-related HCC [33]. Our previous study showed that being overweight and obesity correlated with a more advanced hepatic necro-inflammation and fibrosis in patients with CHB [34]. Previous studies also showed that higher BMI was associated with the risk of HCC development [35]. Cirrhosis status and tumor factors, such as tumor staging, multi-nodularity of tumors, AFP levels and an insufficient surgical safe margin, are well known predictors of tumor recurrence after curative resection [16,17,19]. Consistent with the previous report, our data also showed that baseline cirrhosis status was an independent predictor of early recurrence [36], while multi-nodularity predicts late recurrence after curative resection [19].

Previous studies showed that HBV viral loads and HBsAg levels may predict recurrence after hepatic resection [19,20,37]. In our study, all viral factors, including HBeAg status, HBsAg levels, HBV viral loads and virological response, were not associated with survival. Since about 88% of our patients achieved undetectable HBV DNA within one year after surgery, the impact of these viral factors could be attenuated by NUC therapy. In overall patients, the RFS were comparable between the secondary prevention failure and tertiary prevention groups. Interestingly, in the subgroup analysis, we found that the risk of HCC recurrence was significantly higher in cirrhotic patients with prior NUC secondary prevention failure. Long-term NUC therapy has been shown to improve hepatic necro-inflammation and fibrosis, thus changing the microenvironment favoring hepatocarcinogenesis [38,39,40]. However, in cirrhotic patients who developed HCC despite NUC treatment, the unfavorable tumor biology and microenvironment might not be corrected by controlling HBV, leading to more aggressive tumor behavior. Whether the altered host immune response after antiviral therapy could be responsible for a reduced immunosurveillance for HCC and the underlying mechanisms of the tumor biology in these patients warrants future research to delineate.

A recent study showed that HCC patients with NUC therapy after curative resection generally had preserved liver functions when the tumor recurred, allowing further rescue therapy to improve the outcomes [25]. Consistent with this finding, as 58.3% of patients with HCC recurrence remained in the BCLC stage A in our study, 53.6% of patients could receive second curative treatment. Only 4 patients (4.8%) did not receive rescue therapy after recurrence, and only 1 of them was due to deteriorated liver function. With the benefits of preserving liver functions for further rescue therapy after recurrence, our data supports the general use of NUC after HCC resection.

The 5-year OS rate of 77.3% was also similar to the survivals in previous reports of HCC patients receiving NUC tertiary prevention, and was generally better than those without NUC therapy after surgery [22,23]. Since the 5-year survival rate approaches 80% and most patients who maintained liver function well, the role of host factors that predict tumor recurrence became less significant in OS. In contrast, tumor factors, including BCLC stage, tumor size, microvascular invasion and surgical safe margin were associated with OS in univariate analysis, and microvascular invasion was the only independent predictor of OS. Patients with microvascular invasion had a significantly later tumor stage at recurrence, which may result in a poorer outcome.

Although previous studies showed that NUC therapy could reverse liver fibrosis and cirrhosis [39], it is still unclear whether fibrosis regression occurs in HCC patients. In 10 patients who received a second liver resection in this study, the paired comparison of liver histology showed that half of the patients had fibrosis regression. Since only a few patients received a second hepatic resection and it was difficult to obtain a paired liver histology specimen in the majority of HCC patients, we applied the FIB-4 and APRI as noninvasive indices of hepatic fibrosis [30,31]. At year 2, we observed a significant decrease of APRI, even though the change of the FIB-4 score was not prominent, while significant declines of both FIB-4 and APRI were observed at year 5, including subgroup patients with cirrhosis and those with and without NUC secondary prevention. These data was consistent with previous reports that liver fibrosis may improve, but it required long-term NUC therapy [38,39]. Nevertheless, it should be noted that APRI and FIB-4 may be affected by a change of hepatic inflammation after antiviral therapy [41].

This study has some limitations. First, it is a retrospective study. However, due to the well-established HCC surveillance program and strict reimbursement regulation of NUC prescriptions by national health insurance program in Taiwan, patients received regular follow-up every 2 to 3 months after surgery, allowing close monitoring of virological response and early detection of tumor recurrence. Second, the case number was relatively small in the secondary prevention failure group. However, the risk of HCC development was low in patients receiving NUC therapy, with annual incidence rates of 0.01% to 1.4% in non-cirrhotic, and 0.9% to 5.4% in cirrhotic patients [13]. In the previous nationwide cohort study based on Taiwan’s National Health Insurance Research Database, only 992 out of the 21,595 NUC-treated CHB patients developed HCC between 1997 and 2010 [8]. Therefore the case number in the secondary prevention failure group could not be large even by screening from a large cohort of 512 patients with HBV-related HCC receiving surgical resection in single medical center. Third, patients without NUC treatment after surgery were not included for comparison in this study. However, recent studies have shown that NUC tertiary prevention may decrease the risk of recurrence. Therefore, we only enrolled patients with NUC use after surgery. Since the benefits of NUC treatment still exist in patients with secondary prevention failure, it is not ethical to stop NUC treatment for them despite HCC development. Fourth, the accuracy of FIB-4 and APRI for the prediction of fibrosis stage in CHB was moderate [30,41], and might be interfered with in the presence of HCC. Therefore, this data suggests that these two indices might not be optimal for the prediction of the fibrosis stage, particularly APRI, in patients with HBV-related HCC with and without NUC therapy. Fifth, this study did not include patients with NUC secondary prevention failure who chose radiofrequency ablation or other HCC therapies. Therefore, it is difficult to extrapolate the present finding to state that all cirrhotic CHB patients with secondary prevention failure exhibit a significantly worse RFS than those with tertiary prevention.

In summary, in patients with HBV-related HCC receiving NUC antiviral therapy after curative resection, the RFS and OS were generally comparable between patients with and without NUC secondary prevention. However, cirrhotic patients with NUCs secondary prevention failure had a higher risk of recurrence, and closely monitoring is needed for these patients. Although HCC recurrence may develop despite NUC tertiary prevention, most patients remained in early stage and had preserved liver function during recurrence, allowing a second rescue therapy. Long-term NUC therapy also leads to declines of non-invasive indices of hepatic fibrosis in HCC patients, either in patients with or without cirrhosis or secondary prevention failure.

## Supporting information

S1 Fig Screening, enrollment and grouping of patients. (TIF)

S2 Fig Change of non-invasive indices of hepatic fibrosis from baseline to year 2 in HCC patients receiving NUCs after curative resection (n = 140). (A) Change of FIB-4 scores. (B) Change of APRI.
(TIF)

S3 Fig Change of FIB-4 score and APRI in HCC patients with and without NUC secondary prevention. (A) Change of FIB-4 scores in the secondary prevention failure group. (B) Change of APRI in the secondary prevention failure group. (C) Change of FIB-4 scores in the tertiary prevention group. (B) Change of APRI in the tertiary prevention group.
(TIF)

S1 Table Univariate and multivariate analyses of factors associated with early recurrence within 2 years of surgery in overall 167 HCC patients. (DOC)

S2 Table Univariate and multivariate analyses of factors associated with late recurrence after 2 years of surgery in patients without early recurrence. (DOC)

S1 File Raw data. (XLS)

## Abbreviations

AASLD: American Association for the Study of Liver Disease
AFP: alpha-fetoprotein
ALBI: albumin-bilirubin
ALT: alanine aminotransferase
APRI: AST to platelet ratio index
AST: aspartate aminotransferase
BCLC: Barcelona Clinic Liver Cancer
CHB: chronic hepatitis B
CT: computed tomography
DAA: direct-acting antiviral agents
DM: diabetes mellitus
FIB-4: fibrosis-4 index
HBsAg: hepatitis B surface antigen
HBV: hepatitis B virus
HCC: hepatocellular carcinoma
HCV: hepatitis C virus
HR: hazard ratio
INR: international normalized ratio
MRI: magnetic resonance image
NUC: nucleos(t)ide analogue
OS: overall survival
RFS: recurrence-free survival
