# Supplementary figures and images for: Risk of recurrence in chronic hepatitis B patients developing hepatocellular carcinoma with antiviral secondary prevention failure
Source: PLoS One. 2017 Nov 27;12(11):e0188552. doi: 10.1371/journal.pone.0188552 (PMC5703552; doi:10.1371/journal.pone.0188552)

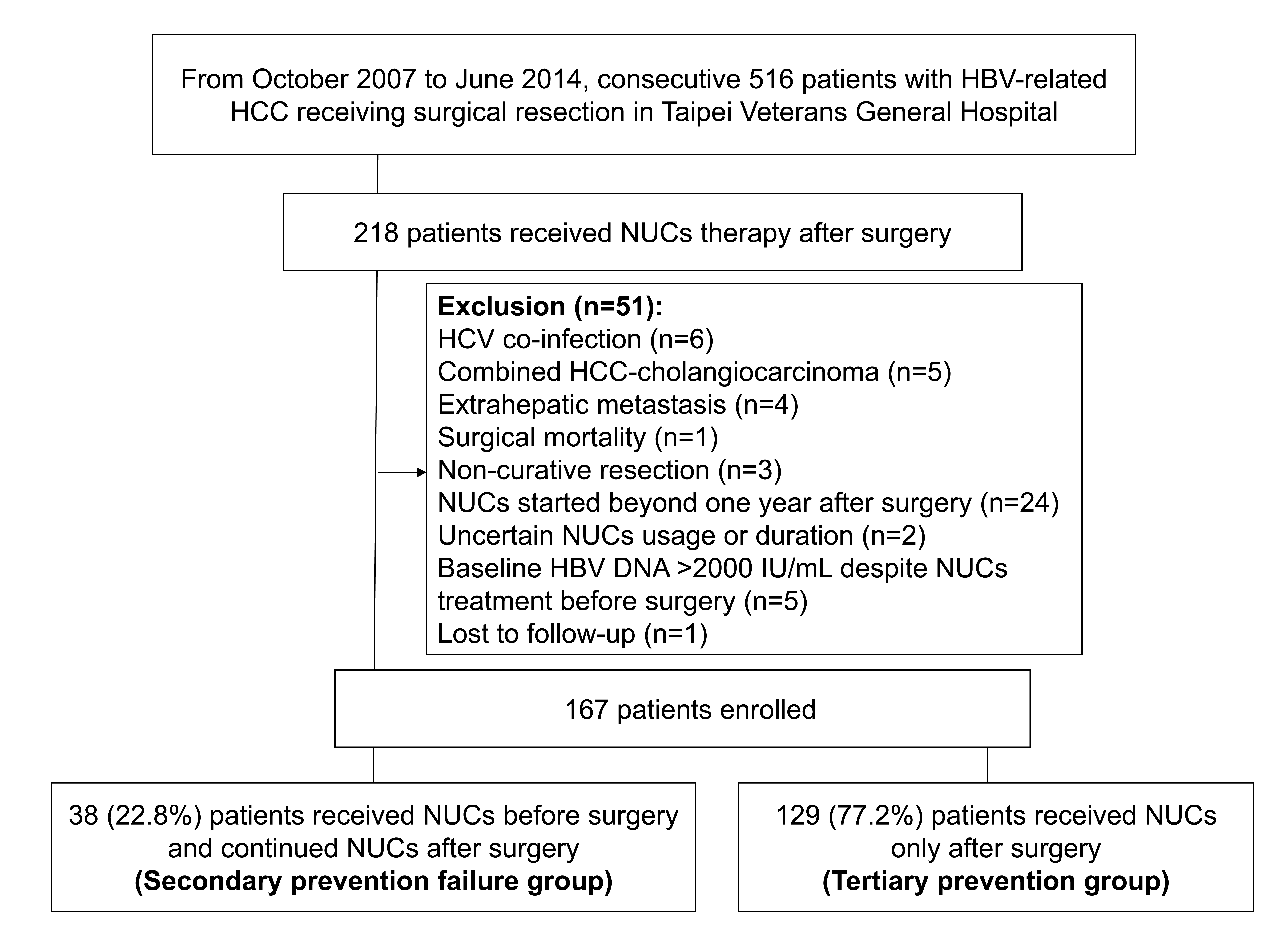

Supplement: S1 Fig — (TIF) [file pone.0188552.s001.tif]
