# Supplementary material for: Risk of recurrence in chronic hepatitis B patients developing hepatocellular carcinoma with antiviral secondary prevention failure
Source: PLoS One. 2017 Nov 27;12(11):e0188552. doi: 10.1371/journal.pone.0188552 (PMC5703552; doi:10.1371/journal.pone.0188552)
Supplement: S1 Table — (DOC) [file pone.0188552.s004.doc]

**S1 Table.** Univariate and multivariate analyses of factors associated with early recurrence within 2 years of surgery in overall 167 HCC patients

|  |  | Univariate | | |  | | Multivariate | | |
| --- | --- | --- | --- | --- | --- | --- | --- | --- | --- |
|  |  | HR | 95% CI | *P* | |  | HR | 95% CI | *P* |
| Age (years) | >60 vs ≤60 | 1.243 | 0.749-2.061 | 0.400 |  | |  |  | NA |
| Sex | Female vs male | 0.364 | 0.089-1.493 | 0.161 |  | |  |  | NA |
| BMI (kg/m^2^) | >27.5 vs ≤27.5 | 2.091 | 1.213-3.605 | 0.008 |  | | 2.185 | 1.206-3.962 | 0.010 |
| Diabetes | Yes vs no | 1.056 | 0.571-1.952 | 0.862 |  | |  |  | NA |
| BCLC stage | B-C vs A | 2.496 | 1.503-4.144 | <0.001 |  | | 2.526 | 1.463-4.360 | 0.001 |
| HBV DNA (IU/mL) | >200 vs ≤200 | 1.015 | 0.563-1.831 | 0.960 |  | |  |  | NA |
| HBsAg (IU/mL) | >200 vs ≤200 | 0.879 | 0.439-1.762 | 0.717 |  | |  |  | NA |
| HBeAg | Positive vs negative | 0.703 | 0.345-1.429 | 0.330 |  | |  |  | NA |
| NUC secondary prevention failure | Yes vs no | 0.889 | 0.481-1.644 | 0.708 |  | |  |  | NA |
| NUC type | High genetic barrier vs low genetic barrier | 1.988 | 0.622-6.350 | 0.246 |  | |  |  | NA |
| Undetectable HBV DNA within 1 year after surgery | Yes vs no | 0.877 | 0.397-1.936 | 0.745 |  | |  |  | NA |
| Tumor size (cm) | >5 vs ≤5 | 2.020 | 1.204-3.390 | 0.008 |  | |  |  | NS |
| Tumor number | Multiple vs single | 1.982 | 1.130-3.478 | 0.017 |  | |  |  | NS |
| AFP (ng/mL) | >20 vs ≤20 | 2.255 | 1.325-3.836 | 0.003 |  | | 2.212 | 1.254-3.900 | 0.006 |
| Bilirubin (mg/dL) | >1.2 vs ≤1.2 | 1.186 | 0.601-2.340 | 0.622 |  | |  |  | NA |
| Albumin (g/dL) | >3.5 vs ≤3.5 | 0.695 | 0.316-1.528 | 0.365 |  | |  |  | NA |
| ALBI grade | Every 1 grade | 1.660 | 0.993-2.776 | 0.053 |  | |  |  | NS |
| Creatinine (mg/dL) | >1.2 vs ≤1.2 | 1.264 | 0.575-2.781 | 0.560 |  | |  |  | NA |
| Prothrombin time (INR) | >1.1 vs ≤1.1 | 1.118 | 0.631-1.982 | 0.702 |  | |  |  | NA |
| Platelet count (10^9^/L) | >100 vs ≤100 | 0.577 | 0.317-1.051 | 0.072 |  | |  |  | NS |
| ALT (U/L) | >80 vs ≤80 | 0.656 | 0.298-1.443 | 0.295 |  | |  |  | NA |
| AST (U/L) | >80 vs ≤80 | 1.561 | 0.812-3.005 | 0.182 |  | |  |  | NA |
| FIB-4 score | >3.25 vs ≤3.25 | 1.934 | 1.160-3.224 | 0.011 |  | |  |  | NS |
| Microscopic vascular invasion | Yes vs no | 2.095 | 1.089-4.030 | 0.027 |  | |  |  | NS |
| Incomplete tumor capsule | Yes vs no | 1.065 | 0.607-1.867 | 0.827 |  | |  |  | NA |
| Presence of steatosis | Yes vs no | 0.957 | 0.552-1.658 | 0.875 |  | |  |  | NA |
| Safe margin >1 cm | Yes vs no | 0.492 | 0.258-0.936 | 0.031 |  | |  |  | NS |
| Histological cirrhosis | Presence vs absence | 2.552 | 1.480-4.402 | 0.001 |  | | 2.989 | 1.655-5.399 | <0.001 |

HR, hazard ratio; CI, confidence interval; NA, not adopted; NS, not significant; ALBI, Albumin-Bilirubin.
