# Supplementary material for: Risk of recurrence in chronic hepatitis B patients developing hepatocellular carcinoma with antiviral secondary prevention failure
Source: PLoS One. 2017 Nov 27;12(11):e0188552. doi: 10.1371/journal.pone.0188552 (PMC5703552; doi:10.1371/journal.pone.0188552)
Supplement: S2 Table — (DOC) [file pone.0188552.s005.doc]

**S2 Table.** Univariate and multivariate analyses of factors associated with late recurrence after 2 years of surgery in patients without early recurrence

|  |  | Univariate | | |  | | Multivariate | | |
| --- | --- | --- | --- | --- | --- | --- | --- | --- | --- |
|  |  | HR | 95% CI | *P* | |  | HR | 95% CI | *P* |
| Age (years) | >60 vs ≤60 | 1.004 | 0.444-2.267 | 0.993 |  | |  |  | NA |
| Sex | Female vs male | 0.039 | 0.000-6.204 | 0.240 |  | |  |  | NA |
| BMI (kg/m^2^) | >27.5 vs ≤27.5 | 0.973 | 0.331-2.856 | 0.960 |  | |  |  | NA |
| Diabetes | Yes vs no | 0.966 | 0.360-2.591 | 0.945 |  | |  |  | NA |
| BCLC stage | B-C vs A | 0.460 | 0.157-1.348 | 0.157 |  | |  |  | NA |
| HBV DNA (IU/mL) | >200 vs ≤200 | 0.800 | 0.314-2.039 | 0.640 |  | |  |  | NA |
| HBsAg (IU/mL) | >200 vs ≤200 | 1.668 | 0.485-5.735 | 0.417 |  | |  |  | NA |
| HBeAg | Positive vs negative | 1.907 | 0.787-4.623 | 0.153 |  | |  |  | NA |
| NUC secondary prevention failure | Yes vs no | 1.542 | 0.635-3.743 | 0.338 |  | |  |  | NA |
| NUC type | High genetic barrier vs low genetic barrier | 1.564 | 0.459-5.333 | 0.475 |  | |  |  | NA |
| Undetectable HBV DNA within 1 year after surgery | Yes vs no | 4.649 | 0.623-34.679 | 0.134 |  | |  |  | NA |
| Tumor size (cm) | >5 vs ≤5 | 0.443 | 0.132-1.488 | 0.188 |  | |  |  | NA |
| Tumor number | Multiple vs single | 2.906 | 1.071-7.887 | 0.036 |  | | 3.028 | 1.112-8.242 | 0.030 |
| AFP (ng/mL) | >20 vs ≤20 | 0.957 | 0.428-2.140 | 0.914 |  | |  |  | NA |
| Bilirubin (mg/dL) | >1.2 vs ≤1.2 | 2.289 | 0.770-6.800 | 0.136 |  | |  |  | NA |
| Albumin (g/dL) | >3.5 vs ≤3.5 | 1.037 | 0.243-4.426 | 0.961 |  | |  |  | NA |
| ALBI grade | Every 1 grade | 1.169 | 0.483-2.829 | 0.729 |  | |  |  | NA |
| Creatinine (mg/dL) | >1.2 vs ≤1.2 | 0.578 | 0.078-4.305 | 0.593 |  | |  |  | NA |
| Prothrombin time (INR) | >1.1 vs ≤1.1 | 3.288 | 1.452-7.447 | 0.004 |  | | 3.359 | 1.480-7.624 | 0.004 |
| Platelet count (10^9^/L) | >100 vs ≤100 | 0.698 | 0.206-2.361 | 0.563 |  | |  |  | NA |
| ALT (U/L) | >80 vs ≤80 | 0.771 | 0.285-2.081 | 0.607 |  | |  |  | NA |
| AST (U/L) | >80 vs ≤80 | 1.597 | 0.594-4.295 | 0.353 |  | |  |  | NA |
| FIB-4 score | >3.25 vs ≤3.25 | 0.713 | 0.243-2.092 | 0.538 |  | |  |  | NA |
| Microscopic vascular invasion | Yes vs no | 0.557 | 0.248-1.251 | 0.156 |  | |  |  | NA |
| Incomplete tumor capsule | Yes vs no | 1.570 | 0.585-4.210 | 0.370 |  | |  |  | NA |
| Presence of steatosis | Yes vs no | 0.843 | 0.294-2.412 | 0.750 |  | |  |  | NA |
| Safe margin >1 cm | Yes vs no | 0.604 | 0.232-1.572 | 0.302 |  | |  |  | NA |
| Histological cirrhosis | Presence vs absence | 1.455 | 0.650-3.261 | 0.362 |  | |  |  | NA |

HR, hazard ratio; CI, confidence interval; NA, not adopted; NS, not significant; ALBI, Albumin-Bilirubin.
